# The Effect of a Moderate Exercise Program on Serum Markers of Bone Metabolism in Dogs

**DOI:** 10.3390/ani10091481

**Published:** 2020-08-23

**Authors:** Zoran Vrbanac, Nika Brkljaca Bottegaro, Branimir Skrlin, Krunoslav Bojanic, Vesna Kusec, Damir Stanin, Maja Belic

**Affiliations:** 1 Faculty of Veterinary Medicine, University of Zagreb, Zagreb 10 000, Croatia; nikabb@vef.hr (N.B.B.); bskrlin@vef.hr (B.S.); dstanin@vef.hr (D.S.); mbelic@vef.hr (M.B.); 2 Ruđer Bošković Institute, Zagreb 10 000, Croatia; kbojanic@irb.hr; 3 Clinical Institute of Laboratory Diagnosis, Clinical Hospital Centre, Zagreb 10 000, Croatia; vkusec@kbc-zagreb.hr

**Keywords:** bone remodeling, biomarkers, exercise, dog

## Abstract

**Simple Summary:**

In this article, we investigate the long-term (four months) effects of a 25 min moderate-intensity treadmill exercise, three times per week, on serum markers of bone metabolism (bone alkaline phosphatase and osteocalcin as bone formation markers and C-terminal telopeptide as bone resorption marker) in dogs. Twenty healthy dogs (Labrador and Golden Retrievers), median age 16.2 (11.7–24.4) months underwent the exercise program. Blood samples were collected as a baseline, mid-term (after two months) and at the end of the study. The values of bone formation markers significantly decreased following two months of exercise program; after which, bone alkaline phosphatase increased while osteocalcin concentration continued to decrease towards the end of the study. Bone resorption marker did not significantly change through the exercise duration. In overall, moderate exercise resulted in no change in bone resorption, and a mild bone formation in young developing dogs.

**Abstract:**

The beneficial effect of physical activity on the musculoskeletal health in dogs is well recognized, but the level of intensity, duration, and frequency of exercise is not fully described. Measurement of serum markers of bone metabolism (bone alkaline phosphatase and osteocalcin as bone formation markers and C-terminal telopeptide as bone resorption marker) during four months of organized moderate-intensity physical training in Labrador retriever and Golden retriever dogs aged between 11.7–24.4 months, showed variations of bone metabolism. Dogs were included in treadmill running sessions for 25 min, three times per week. Blood samples were taken at the beginning of the program (baseline), after two months (mid-term) and at the end of the study after four months. The values of bone alkaline phosphatase and osteocalcin significantly decreased following two months of exercise program. Bone alkaline phosphatase increased by the end of four-month training cycle, but did not reach baseline value. Osteocalcin levels continued to decrease towards the end of the study. C-terminal telopeptide concentrations did not significantly change throughout the study duration. The results of this study show that aerobic exercise of moderate-intensity caused an initial decrease in bone formation followed by an increase of bone alkaline phosphatase and a further decrease of osteocalcin concentration. The response of two formation markers can be explained by the different stage of osteoblast activity that they express. In summary, moderate exercise resulted in no change in bone resorption, and a mild bone formation in young developing dogs.

## 1. Introduction

Bone remodeling is the basis of bone tissue development, which involves osteoclast degradation, reversal period, and bone formation under the influence of osteoblasts. Osteoblasts and osteoclasts synthesize substances that can be measured in the blood or urine, and some of them are of clinical importance in assessing bone metabolism.

Bone formation markers commonly used are bone-specific alkaline phosphatase (BALP), osteocalcin (OC) and amino and carboxy propeptides of collagen type I (PINP and PICP). Bone resorption markers include pyridinoline (PYD), deoxypyridinoline (DPD), enzyme tartrate-resistant acid phosphatase (TRAP), and amino and carboxy telopeptides of collagen type I (NTX and CTX). To monitor the remodeling activity, one indicator of bone formation and one of bone resorption is usually determined. Since there are daily fluctuations and the influence of other factors in humans and animals [1,2], blood or urine samples are recommended to be always taken in the morning at the same time [3]. Quantitative measurement of biochemical parameters of bone remodeling by immunoenzymatic (ELISA) or radioimmunoassay (RIA) methods is performed with commercial reagent kits. The applicability of using human tests in veterinary medicine has been established in previous research [4,5].

In dogs at rest, the activity of biochemical indicators of bone remodeling in serum, the differences with regard to age, sex, size, breed, the influence of hormones and daily variability have been investigated [3,6,7,8,9].

The impact of physical activity on bone mass is mediated by bone remodeling dynamics and hormones associated with the skeletal system, although not all mechanisms involved have been fully elucidated [10,11]. The investigated influence of long-term physical activity on bone metabolism in humans has given ambiguous results on changes in biochemical parameters of bone remodeling. Bone formation markers BALP and OC increased in a study after five weeks of endurance exercise [12], yet a decrease was noted in another after four weeks of aerobic exercise [13]. After one year of exercise (walking and walking/jumping groups), BAP was increased, but OC was not changed significantly [14]. A decrease in bone resorption marker was observed after six months of high-intensity resistance training [15] and four weeks of aerobic exercise [13], but no changes after 12 month low-repetition jump training [11], 26 months of intense exercise [16], six months of low-repetition high-impact training [17], and 12 months of exercise [14]. Current knowledge in veterinary medicine on the impact of physical activity on bone metabolism by measuring biochemical parameters of bone remodeling is limited. Study on dogs in the phase of intensive growth that started running from the age of 15 weeks for one year [18] showed no correlation with bone markers and observed changes in bone mineral density (BMD). In young horses the effect of training over 14 weeks [19], and 20 weeks [20], showed that despite the increase in BMD, due to training, bone remodeling was reduced, i.e., equalized with the physiological values of the appropriate age at the end of the experiment. Physical conditioning of young horses during 90 days showed OC levels to be declining linearly [21] After a 7 km race in adult greyhounds, both OC and PYD were not changed from baseline values, while BALP increased [22]. Studies of the effect of long-term moderate-intensity physical activity on changes in biochemical parameters of bone remodeling in adult dogs have not been reported. A better understanding of biochemical indicators of bone remodeling can determine their possible application in clinical veterinary practice, i.e., determining the intensity of therapy and monitoring the success of rehabilitation.

The aim of the study was to investigate the effects of a moderate-intensity treadmill exercise on blood biochemical parameters and bone markers (BALP and OC as bone formation markers and CTX as bone resorption marker) in 20 healthy dogs.

We hypothesized that bone remodeling will be accelerated by the effect of organized physical activity relative to baseline values.

## 2. Materials and Methods

### 2.1. Animals

Twenty-two dogs were enrolled in the research protocol. The inclusion criteria were unremarkable physical and orthopedic examination as well as CBC and serum biochemistry findings. One dog was excluded due to kidney disease detected after the baseline exam, and another one dropped out of the training program due to unbalanced behavior. Only 20 dogs completed the study: Fifteen Labrador Retrievers and five Golden Retrievers. Fourteen dogs were females and six were males. The median age of dogs was 16.2 (range 11.7–24.4) months, and their median weight was 30.4 (range 22.8–39.4) kg. All dogs were neutered as six months old. The dogs were divided into two groups, under 18 months of age and over 18 months of age, each group consisting of 10 dogs. The division in groups was based on the assumption that the skeletal growth for 18 months old is completed. The median age of the younger group was 13.7 (ranging from 11.7 to 14.4) months and 21.0 (ranging from 18 to 24.4) months in the older group, and the median weight was 28.1 (range 22.8–39.4) and 31.4 (range 27.9–37.6) kg, respectively. The dogs were in the program of suitability assessment for guide dogs. Their inclusion in this study under the ethical and animal welfare standards was approved by the Institutional committee (File no. 640-01/11-17/39, Record no. 525-06-1-0255/11-3).

Dogs were housed in kennels (205 × 240 cm) built under International Guide Dog Federation Standards [23]. They were fed the same super premium food (Eukanuba Professional, Adult, IAMS Europe, Coevorden, Netherlands) with freshwater available ad libitum. Energy requirements were calculated individually using the formula 110 kcal ME/kg^0,75^ [24]. Dogs weights were recorded on a weekly basis, and energy intake was adjusted to ensure that that the dogs maintain their body weight. The amount of daily activity apart from designed supervised exercise protocol was the same for all animals, it included three sessions of 15 min free activity in the running area and one-hour service and guide dogs training program.

### 2.2. Exercise protocol

The exercise protocol was established on the basis of a moderate treadmill exercise (Fit Fur Life Ltd., Professional model, Surrey, UK) for each animal individually, with constant heart rate HR monitoring (Polar WearLink, Polar RS800CX, Electro Inc., Kempele, Finland) [25]. Dogs were previously untrained, so the adjustment period to the treadmill and wearing a heart rate monitor began two weeks prior to the beginning of the study. It included 5 min warm-up phase, gradual incremental speed period until the desired HR was reached, 20 min exercise on that speed, and a 15 min recovery phase. The exercise intensity was set up individually to HR equivalent of 50% of predicted HRmax. The HR ranged 129–142 beats per minute. The average speed was 7.45 km/h. During a 4-month period, the animals were trained three times per week for 25 min. All safety measures were undertaken to prevent any injury.

### 2.3. Sample collection

Blood samples were obtained three times from all dogs (at the beginning, after two months and at the end of four-month period). The samples were taken from *venae cephalicae antebrachia* into vacuum tubes for serum with gel using a vacutainer (Vacutainer^®^, Becton Dickenson, Franklin Lakes, NJ, USA). Due to daily variations and circadic rhythm previously described [3,26], blood samples were taken 24 h post exercise in the morning between 9–11 am. After the collection, the tubes were centrifuged for 15 min at 3000 pm and serum was stored at −70 °C.

### 2.4. Assays for the bone markers

The bone markers selected for this study were: Bone alkaline phosphatase (BALP) and osteocalcin (OC) as indicators of bone formation, and C-terminal telopeptide of type I collagen (CTX) as marker of bone resorption.

The activity of BALP was measured using a commercial immunoassay kit (Metra^TM^ BAP EIA kit, Quidel Corp., San Diego, CA, USA). The concentration of OC was determined by using a commercial kit (N-MID^TM^ Osteocalcin ELISA Nordic Bio-science Diagnostics, Herlev, Denmark). The concentration of CTX was determined by using a commercial kit (Serum CrossLaps ELISA, Nordic Bio-science Diagnostics, Herlev, Denmark). Assays used previously demonstrated to have good cross-reactivity in canine research [6,7,9].

Serum biochemical parameters were determined using Olympus AU 600 (Olympus diagnostica GMBH) and included: glucose, alkaline phosphatase (ALP), aspartate aminotranspherase (AST), alanine aminotranspherase (ALT), creatine kinase (CPK), lactate dehydrogenase (LDH), urea (BUN), total proteins, albumins, cholesterol, triglycerides creatinine, magnesium, and calcium.

### 2.5. Statistical analyses

All exploratory and statistical data analyses were performed using Rstudio software version 1.2.5033 as an environment for R v3.6.1 (R: A language and environment for statistical computing. URL: https://www.R-project.org/). Biochemical parameters were analyzed as dependent variables and times of observation, age and sex as independent explanatory variables. Univariate analyses for the association of the times of observations with dependent variables were performed using paired Student’s t-test for normally distributed data with Welch correction applied if variances were unequal. For non-normally-distributed data paired Mann-Whitney rank sum test was performed. Associations of categorical demographic variables were analyses using ANOVA if data complied with test assumptions and if not, using Kruskal-Wallis test. In order to account for repeated measurements at the dog subject level, multivariate analyses were performed using generalized linear mixed effects models with times of observations, age, and sex as fixed effects and dog subjects as random effects. Model building was performed by backward step procedures and model selection using ANOVA tests. Models not complying with the test assumptions were retested using other error distributions (e.g., lognormal, gamma, etc.) or non-parametric tests if test assumptions were still not satisfied. The level of significance was set at less than 0.05 for all univariate and multivariate analyses. Correlations between biochemical parameters were performed using Spearman’s rank correlation tests with Bonferroni adjustment of *p* values.

## 3. Results

**Figure 1:**
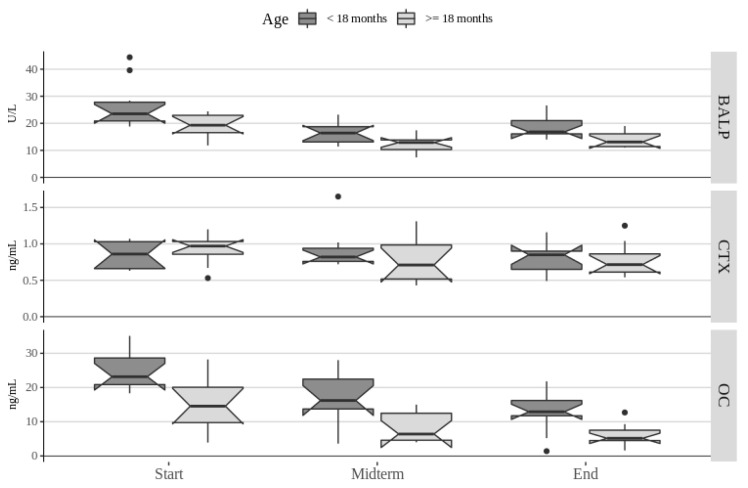
Distribution of bone remodeling biomarkers in 20 dogs of two equal-sized age groups during a four-month long moderate-intensity exercise program. The bold bar shows the median value, the lower and upper hinges correspond to the first and third quartiles, and the whiskers extend from the hinges to the largest/lowest value no further than 1.5 times the inter-quartile range from the upper/lower hinge. Data beyond the end of the whiskers are outlying points and were plotted individually. Overlapping of notches between different boxplots is indicative of no significant difference between respective groups. Abbreviations: bone alkaline phosphatase (BALP), C-terminal telopeptide (CTX), and osteocalcin (OC).

Pairwise comparisons of parameter values between times of observation showed significant differences in values of OC, BALP, ALP, calcium, total proteins, albumin, BUN, creatinine, triglycerides, cholesterol, glucose, but no significant difference in values of CTX, LDH, and CPK. Changes in bone remodeling markers by age during the course of the study are presented in Figure 1.

The concentration of OC had a consistently significant decrease during the course of the study. The average values of BALP showed a significant decrease at mid-term from the baseline values that thereafter significantly increased but still remained significantly lower from baseline values at the end of the study. The concentrations of albumin, cholesterol, total protein, creatinine, and calcium significantly decreased from the baseline values at both mid-term and at the end of the study, but the differences between mid-term and end of the study were not significant. Conversely, the concentrations of ALP, BUN, AST, glucose, and triglycerides were significantly increased by the end of the study, compared to both the baseline and mid-term values, but the increase was delayed as the mid-term values were not significantly different from baseline values. Magnesium values were significantly different only between baseline and mid-term of the study and ALT values were not significantly different only between the end of the study and baseline values.

Univariate analyses of age and sex also showed significant associations with several parameters of interest. Throughout the study, BALP and OC concentrations were significantly associated with age. Magnesium, total protein, and creatinine values were observed to be significantly associated with age at baseline and mid-term, whereas ALT, CPK, LDH, ALB, glucose, and urea were significantly associated with age at least at one of the measurements during the study.

The final multivariate model for OC showed significant associations with times of observation (*p* < 0.001) and age (*p* < 0.001) as independent predictors. Holding other covariate constant, OC values in dogs under 18 months of age were on average by 8.4 IU/L (95% CI 4.0–12.8) lower than in older dogs, while values at mid-term were by 7.9 IU/L (95% CI 5.4–10.3) and at the end by 10.6 IU/L (95% CI 8.2–13.1) lower than baseline values but with no significant difference between values the mid-term and end of the study. Similarly, BALP values were significantly associated with times of observation (*p* < 0.001) and age (*p* < 0.001) showing an average decrease of 4.9 IU/L (95% CI 2.8–7.1) in dogs younger than 18 months of age compared to older dogs, and with regard to times of observation, a decrease of 8.0 IU/L (95% CI 5.2–11.0) at mid-term and 6.3 IU/L (95% CI 3.4–9.3) at the end of the study compared to baseline, but with no significant difference between values at mid-term and at the end of the study.

Age and times of observation showed significant associations with total protein concentrations and only age with magnesium concentrations. Total proteins were on average higher by 3.8 g/L (95% CI 0.5–7.1) in dogs older than 18 months of age compared to younger dogs, and were lower by 2.6 g/L (95% CI 0.1–5.1) and 3.1 g/L (95% CI 0.6–55) at mid-term and the end of the study compared to baseline values, respectively, while not being significantly different between mid-term and the end of the study. Magnesium was higher by 0.05 IU/L (95% CI 0.001–0.1) on average in dogs older than 18 months of age compared to younger dogs.

Multivariate analyses of ALP, ALT, AST, glucose, BUN, creatinine, albumin, and calcium returned no significant associations with age and sex. 

The significant difference of parameter average values between times of observation agreed with results of univariate analyses.

## 4. Discussion

The obtained results of bone markers activity during four months of moderate treadmill exercise partly confirm the hypothesis, since bone resorption marker did not significantly change and only one bone formation marker showed changes that can be attributed to exercise impact.

All measured basal values for OC, BALP and CTX are in the range of reference values described so far in dogs [6,7]. However, when taking into account age groups, the basal OC concentration and BALP activity of the studied group of dogs corresponds to the described values in dogs of the same breed aged 18 months [27], but appear slightly higher than the reference values for that age described in dogs of various breeds [4,6]. Skeletal growth and development in young animals are manifested by high dynamics of bone remodeling, in which construction predominates [28]. Guided by these reference values, the present sample would correspond to the age group of dogs in which bone formation is still pronounced. It decreases with age in dogs [6,7,27]. Osteocalcin concentrations reach their maximum during puberty and decline to adult levels [22,29].

After two months of programmed training, the obtained results indicate that there was a significant decrease in the values of the measured parameters of bone formation of BALP and OC compared to the initial measurement. The concentration of CTX also decreases, although not significantly.

In general, the decrease in the values of both indicators of bone formation could be explained by the fact that at the time of measuring the initial values of these parameters bone activity of the young organism was at its peak, especially in the younger group, and the measured values reflect the still great dynamics of bone metabolism at that age. In this direction, Sanecki et al. [30] noted that the activity of BALP decreases towards the fifteenth month of the dog’s age, when it approaches the values in adult dogs. Given that the average age of the dogs in this study was 16.2 months at the start of the study, the measured sharp decline in BALP activity can be explained by physiological processes of appropriate age.

Another explanation for the results suggesting that after two months of moderate-intensity programmed physical activity there was decreased bone activity in terms of bone formation may be similar to changes reported in several studies in humans and horses that suggests a skeletal adjustment reaction to the new situation. Namely, for the dogs used in this research, entering the training program was an additional, new physical activity that they had not encountered before, i.e., a new form of mechanical stimulation on their body, and thus, the adaptation response is expected. Running on a treadmill is different from running freely, which was practiced by dogs in our study until then, so the very surface of the moving treadmill requires adjustment. Untrained horses placed in training showed a decrease in osteocalcin concentration [20,21,31]., followed by an increase at a later stage of training [31].Similarly, after four weeks of aerobic activity in humans, a significant decrease in osteocalcin concentration [32], and osteocalcin and bone alkaline phosphatase [13] was reported.

Decreased values of all three biochemical indicators of bone remodeling after two months of physical activity in this study are indications of a decrease in bone remodeling in general, and not specifically indicators of degradation or construction. In still-developing young animals, physical activity may reduce the extent of bone deposition or metabolism in general [33]. McCarty and Jeffcott [33] reported lower osteocalcin concentrations in horses trained on the treadmill than in control groups, although the value itself increased from baseline in both, suggesting that the exercise program had a negative effect on osteocalcin concentration. In this study, the concentration of osteocalcin continued to fall, and there was a statistically significant difference between all three measurement points, i.e., there was a linear decline from the initial measurement towards the end of the four-month period of activity.

At the end of the study, BALP activity was significantly lower than initially measured, but was significantly higher than the midterm value in both groups. Roghani et al. [34] reported significantly increased BALP activity in the group of women who underwent a six-week moderate-intensity running program, 30 min per day on a treadmill.

The differences in the direction of changes in the values of the two indicators of bone formation, bone alkaline phosphatase and osteocalcin, can be explained by the fact that their values are associated with different stages of growth and differentiation of osteoblasts. It is possible that BALP, as an isoenzyme found in the osteoblast membrane, has a different response to exercise-induced stress than OC, which is mainly associated with the late phase of bone mineralization [35,36]. Namely, OCis probably involved in the later phase of mineralization rather than in the synthesis of the organic matrix. As matrix synthesis precedes mineralization, the bone formation may have begun before OC increased. Osteocalcin is not detected during osteoblast proliferation in vitro, its concentration increases only with the accumulation of total mineral [37].

According to Banfi et al. [2], the shift of BAP values towards baseline values, i.e., the increase after four months of physical activity compared to the previous measurement, was caused by aerobic exercise in our study, given that similar studies in humans showed that bone alkaline phosphatase is more sensitive to aerobic training and osteocalcin to anaerobic training.

Although the concentration of C-terminal telopeptide of type I collagen, measured after two and four months of the experiment, has a slight downward trend, no statistically significant difference was found between the three measurement points. The concentration in all three measurements corresponds to the reference values described in dogs younger than two years. This result for the CTX in humans was described by Eliakim et al. [12] in whose study young men aged 15 to 17 exercised two hours a day for five weeks. The concentration of CTX did not change significantly in their study, although BALP and OC increased. Another study in a group of young people showed that aerobic physical activity caused a statistically significant decline in bone resorption rates relative to baseline values [13].

High-Intensity physical activity leads to changes in C-terminal telopeptide type I collagen concentration [15] as it represents a part of bone collagen that is released during the breakdown of bone into serum and it is considered as an indicator of bone resorption. [38]. Since in this study, dogs participated in programmed moderate-intensity physical activity, no significant decrease in CTX concentration observed could be explained by lower exercise intensity.

Statistically significant and positive correlations between total proteins and albumins with magnesium and calcium were expected because both of these minerals have to fraction in the blood which binds to albumins, i.e., proteins. The positive correlation between magnesium and calcium can be explained by the fact that both minerals participate together in the majority of functions, such as taking part in the process of energy metabolism, assisting the maintenance of normal muscle function and heart function, conducting nerve impulses, bone turnover, etc., and as mentioned above, both minerals are bound to the same transporters in the blood, albumins [39,40].

Between the ages of 13 and 15 months, BALP activity falls sharply and reaches normal values for adult dogs [5] Hence, a lower value of bone alkaline phosphatase after two months corresponds to this physiological reason, and the increase after four months of activity could be described as a positive impact of programmed physical activities. Namely, we would expect that the activity of BALP will continue to fall or will remain at the previous value, so a statistically significant increase can be attributed to the influence of mechanical load, due to physical activity. The increase in BALP activity did not reach or exceed baseline values, similar to other studies in young horses [41] and males [13].

Authors are aware that the lack of a control group is a limitation of the study. However, the observed changes in BALP activity overall and especially in the younger group, are indicative of the impact of training on bone activity.

## 5. Conclusions

The results of this study show that aerobic exercise of moderate-intensity caused an initial decrease in bone formation with consequent increase of BALP and a further decrease of OC concentration. Age and skeletal maturity, as well as the physical activity program, are critical factors that must be considered when assessing the impact of physical activity on bone remodeling.

The results also suggest the importance of selecting at least two different bone formation indicators given that each corresponds to a different stage of bone cell function and because a certain exercise intensity, as well as daily fluctuations, can affect the outcome of the measurement.
